# Risk, Responsibility, Rudeness, and Rules: The Loneliness of the Social Distance Warrior

**DOI:** 10.1007/s11673-021-10135-x

**Published:** 2021-10-25

**Authors:** David M. Shaw

**Affiliations:** 1 Institute for Biomedical Ethics, University of Basel, Basel, Switzerland; 2 Care and Public Health Research Institute, Maastricht University, Maastricht, The Netherlands

**Keywords:** COVID-19, Coronavirus, Rules, Responsibility, Decision-making, Public health ethics

## Abstract

We have a responsibility to obey COVID-19 rules, in order to minimize risk. Yet it is still seen as rude to challenge people who do not respect those rules, when in fact the opposite is true; it is rude to increase risk to others. In this paper I analyse the relationship between risk, responsibility, and rudeness by analysing the evolution of the main governmental slogans and rules and explore the complex relationship between simplicity, safety, and perceived fairness of these rules, and how these features in turn influence the extent to which we act responsibly. I begin by exploring the relationship between rudeness and risk in our interactions about coronavirus, before going on to analyse the importance of clear rules in minimizing tension between us, illustrating the argument with various slogans including “stay at home,” “stay alert,” and the now infamous “rule of six,” which is actually at least three different rules. Ultimately, we are faced with a paradox: people annoyed about complex/unfair rules are less likely to obey them, even if that means rules will apply for longer and even though it was noncompliance with earlier simpler rules that means new rules are necessary. And if rules make less or no sense it is harder to try to get people to follow them in your own capacity as a citizen; it is hard to police rules that are seen as arbitrary or unfair.

## Introduction: The Loneliness of the Social Distance Warrior

Coronavirus has changed the way we interact with one another. No more hugs (except with children, in Scotland at least); cross the road to avoid others; wear a facemask. But some people are better than others at assimilating all the new obligations that are conveyed to us through various rules and slogans. This in turn means that our relationships are also changed by the fact that it is sometime necessary—or at least desirable—to draw someone’s attention to the fact that he or she is breaking or bending one of the rules. Unfortunately, anyone who does “whistleblow,” even politely, runs the risk of being seen as rude.

Here is an example. In the context of coronavirus, “Don’t stand so close to me” is a message that most of us have internalized and try to bear in mind whenever we are out and about. But even with the lifting and reimposition of lockdown restrictions, many people still forget that physical distancing is very important; when we had relatives visiting our house recently, I found this out for myself. This episode occurred when household visits in Scotland were still permitted, before the autumn restrictions were brought in.

On the first day, it was our daughter’s birthday and we had some relatives over for a mini-party (in line with the restrictions at force at the time). We tried to respect physical distancing as much as possible, but one couple didn’t sit together as planned, and at one point one of them—let’s call him Jack (not his real name)—asked to hold my phone to look at a photo, which isn’t advisable in terms of social distancing. These were very minor issues that attracted no direct comment, though he did look a bit grumpy when I refused to hand over my phone and showed him the picture instead. I remarked to my wife that some of our guests seemed to be starting to forget the importance of physical distancing.

The next day we had visitors again. We were going to have a family dinner and had already decided that we would not sit at the table together but would instead disperse to eat in order to avoid sitting too close to each other. At one point before dinner I was sitting at a table playing a game with the kids, when I realized that Jack was standing right next to me, so close that he was almost touching. I immediately said “give us some space please.” Jack did not budge, except to glare at me from a very close distance. I repeated my request. He sighed, made a pained expression, then took a very small step to his left away from me—staying well within one metre. Raising my voice a little, I then said “take social distancing seriously” and he took another step. Then he went straight to the kitchen where he complained that I had been rude to him.

Was I rude to him? All I did was politely request that he respect the physical distancing rule and then repeat that request twice (and I would have moved myself if I hadn’t been playing a game with the kids). If anything, he was rude to me—at least three times. First, it is the height (and breadth) of rudeness to stand right next to someone during a pandemic. Second, it is antisocial to ignore a request to respect social distancing. Third, it is rude to mock such a request by making a joke of finally (and grudgingly) complying. It also seems pretty rude to then go and complain that you’ve been treated rudely. Of course, the rudest thing of all for me to do would have been to say nothing and risk infecting Jack with COVID-19.

Worse was yet to come: I was then accused by my wife of being rude to Jack. I replied along the lines of the previous paragraph. She replied that I was taking the rules too seriously, as “he hasn’t been out anywhere, you’re not going to get the virus from him.” But that wasn’t why I asked him to move away. I did that to avoid him getting infected by me, as I’ve been out quite a lot and he’s in a higher-risk group. Also, given his disregard for distancing, a reminder of the rules might decrease his chances of getting infected elsewhere. (Actually, given that Jack is relaxed about physical distancing and had spent a few days in close proximity to people visiting from England, where prevalence was higher than in Scotland, it’s quite possible that he was at greater risk of infecting me than I was him).

Reminding someone of rules might seem condescending despite the best of intentions, particularly given differences in perceived familial seniority. To some extent, I was “getting in Jack’s face” by asking him not to get in mine and could have been seen as taking the rules too seriously. But there is a high risk of people having COVID-19 and not knowing it and in turn inadvertently leading to the infection of one of us or someone not present in our interaction; the harms of not taking the rules seriously are morally distant from such encounters, making the reminder of the rules seem ruder than it actually is. An additional point is that, when people are trying to relax in social situations, reminders of the reality of the pandemic may be unwelcome to say the least, with the reaction that the messenger might be accused of being rude even if the message is not really.

The role of social distance warrior can be a difficult one, particularly so in Britain, where so much importance is placed on politeness. But it is clearly a whole lot ruder to run the risk of infecting someone with a disease that could kill them than to ask them to keep a safe distance. In the end, my wife agreed that I had done nothing wrong, but that isn’t quite the same as saying that I did the right thing. (It’s a dirty job but someone has to do it.)

## Simplicity, Safety, and Fairness

How can the perceived rudeness of insisting on social distancing be overcome? One potential means of doing so is to hammer home the importance of such measures through effective messaging. If Jack has a simple slogan to remember, it might help avoid such awkward situations from developing. However, analysis of the various rules in force across the United Kingdom reveal that simplicity often comes at the cost of logic, safety, and fairness, undermining the aim of making the message simple in the first place.

Initially, the slogan across the United Kingdom was very simple and memorable: “Stay at home” (UK Government 2020a). This was also very accurate, as the basic public health message of lockdown was to remain in your home at all times except for one period of exercise per day and any required shopping (or medical) trips. This simple message was also very safe, as social distancing was largely accomplished during this period by keeping walls between people, rather than two metres of space. Evidence suggests that people generally understand and try to comply with social distancing rules and messages, even if some—like Jack—might forget them occasionally (Benham et al. 2021). However, the message has become more complicated because initial lockdown was simple by necessity; the more lockdown loosens, the more complex rules become, making it harder to communicate them; complexity introduces confusion and perceived conflict between different public health messages, with the result that some members of the public think that a given rule does not make sense; one example is the one-metre distancing requirement for certain contexts and the two-metre requirement elsewhere (Benham et al. 2021).

The problems caused by increasing complexity were made abundantly clear by the shift (in England) to the new slogan “Stay alert.” Like “stay at home,” this is a simple slogan; unlike “stay at home,” it is not a simple message because it is highly ambiguous. The new message was widely criticized for being unclear and for giving the impression that one can stay alert in order to defend oneself against an imperceptible virus. This is clearly not effective public health communication, yet one can see the motivation for it; a new slogan was needed because people no longer had to stay at home, and if people are mixing more they do not to stay alert in terms of respecting physical distancing rules. In that sense, “stay away” would have been more useful, if perhaps a little too alarming. (Unfortunately, the U.K. government paid for advertising on social media targeted at all U.K. residents, meaning that people in Scotland and elsewhere received false information about the loosening of restrictions (Shaw 2020a).)

It had seemed likely that the U.K. government would instead adopt “stay safe” as its slogan, which might have been a good compromise between the active but vague stay alert and the precise but alarming stay away. “Stay safe” suggests keeping a safe distance (implying stay away) while also suggesting that you need to remember to do that. Indeed, “stay safe” has become a common farewell utterance during the pandemic, though it does have some potentially sinister undertones (Shaw 2020b).

Somewhat ironically, perhaps the slogan that saw the greatest uptake by the public was not “stay alert” but “eat out to help out” (U.K. Government 2020b), which encouraged people to dine out at a discount, leading to a resurgence in viral transmission. This new slogan totally eclipsed another public health message that launched in England the same month: “hands, face, space” (U.K. Government 2020c). In Scotland, the acronym FACTS has been repeated regularly but has not caught on. This is perhaps unsurprising given that its full meaning is: “Face coverings; Avoid crowded places; Clean your hands regularly; Two metre distance; Self-isolate and book a test if you have symptoms” (Scottish Government 2020a). This is clearly far too complicated, and “hands, face, space” a much more effective *aide-memoire*; had this caught on instead of eat out to help out, it might well have helped reduce transmission.

Perhaps in recognition of the ambiguous messaging of “stay alert,” in September 2020 there was a renewed focus on simplicity with the unveiling of the much-heralded “rule of six.” This meant that a maximum of six people from six households could meet, either indoors or outdoors. This is quite a simple message and fairly memorable. However, there are two issues with it. First, the rule of six in Wales and Scotland is different from the rule of six in England; second, by aiming for simplicity, it potentially sacrifices safety.

In Scotland (Scottish Government 2020b) and Wales (BBC 2020), the rule of six meant that six people could meet six people outdoors or indoors, as in England. However, these people must be from a maximum of two households. Thus, though a person living alone in England could have five friends each of whom lives in a different house round for a coffee, in Scotland only one such person could come round. This is safer than the English rule as it gives the virus less chance to spread but also less simple because it adds into the equation the number of households as well as the number of people. In turn, it raises the issue of perceived fairness; why should people in Scotland only be allowed one household to visit their home, when in England it could be as many as five? The latter is much more permissive, and if it is safe, it seems unfair not to apply the same rule in Scotland. Such perceived unfairness is also a safety issue, because if a rule seems unfair people are less likely to follow it.

Another difference between England and Scotland/Wales concerns children. Initially, children under twelve were exempt from the maximum number of people in Scotland (and eleven in Wales), meaning that larger groups than six could mix if some of them were the right age. This seems fairer than the situation in England and does not increase risk substantially. However, children were not exempted from the household limit, making the rule even more complicated. The exception for children under twelve does make sense but was not carried to its logical conclusion; why could it not be (at least) six kids from different households who could meet? The Scottish rule meant that two families with two adults and five children each can meet indoors or out, but that a child could not even have two friends (from different households) round for a play, much less a birthday party. However, within a matter of weeks this incongruity was recognized and children were also removed from the number of households, meaning that birthday parties became possible again—in Scotland at least.

The intertwined issues of safety and fairness are also in tension when we consider the rule of six’s institutionalization of equivalence between indoor and outdoor gatherings. Even at an intuitive level this seems odd, as for many months the public have been told that seeing people outdoors is much safer than seeing them indoors because the virus spreads much less easily in such contexts. It is much simpler to have the same rule for inside and outside, but it doesn’t seem logical, given that the science hasn’t changed. If it is still the case that meeting outside is safer, then either the outdoor limit has been artificially lowered to the indoor limit, meaning that the rule of six is unfair by virtue of being arbitrary (to some extent) or the indoor limit has been unsafely raised to six to match the outdoor level. Either way, all these issues increase confusion and the risk of noncompliance. (The rule of six was only in force in Scotland for two weeks before all indoor gatherings were banned in Scotland (Traynor 2020), again reinforcing the relative safety of the interior and exterior environments and also highlighting the extreme speed with which regulations can change—rapidity that further increases confusion regarding which rules are in force.)

## Rudeness and Rules

It might seem as if rudeness isn’t really affected by or related to the simplicity, safety, or fairness of rules. But in fact it is very closely linked; if the rules don’t (seem to) make sense, you will be seen as rude(r) for trying to enforce them, particularly if you see why they make sense and the person who isn’t following them doesn’t. If you say, as part of your attempt at getting someone to follow the rules, that the rules make sense because X (perhaps in response to a claim that they don’t make sense), you will be seen not only as a busybody but also as a condescending one. However, it is a sad fact that rules do tend to make sense scientifically even if some people think they don’t; if someone has a cough, telling them to respect COVID-19 rules might seem reasonable, but if they don’t, their moral distance from the consequences of getting or passing on COVID-19 might make you seem ruder. Thus the perceived rudeness of COVID-19 rule-reminding is heavily context-dependent; it relates not only to the perceived logic of rules but also to the relationship with the person doing the reminding, the context of the conversation, and the moral distance from the potential harm of any rule breaking.

Some examples of rules that are perceived as not making sense will be helpful here. One example given by outraged (mainly Conservative) politicians was the restriction on music in pubs. This might be seen as a paradigm of a police state: the people can’t listen to music while enjoying themselves. But of course, the logic behind this ban is very clear: if people have to raise their voices, which they are likely to have to do in a noisy pub, that increases the risk and range of any virus spread.

Another example is frustration at the ban (in some parts of England and all of Scotland) on households mixing in homes. “Why,” asks the cynic, “can we not have friends in our houses, yet we are free to get intoxicated in a public bar?” Once again, however, the logic is clear: in pubs, there is a degree of regulation and oversight; care can be taken to ensure that physical distancing is respected and that households are not mixing too closely. Within homes, there is no such oversight, and the evidence shows that private gatherings are the source of many infections.

This brings us on to the third example: “why are they introducing rules banning household visits and reducing pub opening hours when the virus is spreading in schools?” This is wrong both factually and in terms of the logic of the rules. The virus isn’t spreading (much) between school pupils and we would know if it was because of contact tracing. It is spreading a little between teachers but only when physical distancing is not followed. So the rule makes sense but won’t seem to make sense to anyone who doesn’t understand virus vectors and contact tracing—which is unfortunately a large segment of society.

Because of all these misunderstandings, and the aforementioned tensions generated by rules that aim at simplicity but seem unfair or contradictory, many members of the public are simply giving up. “There’s no point in following the rules” and “if we’re going to get it we’re going to get it” being two of the prime examples of this attitude. But of course, if the original rules when lockdown was loosened had been followed, all these new rules would not be necessary—and if people don’t follow the rules, things will get even worse next time. A U.K. study found that members of the public believed clearer communication of the scientific rationale behind rules would increase compliance (Benham et al. 2021).

Another example of a common reaction is the idea that “it’s time to let people risk assess their own lives.” Claims like this are somewhat ironic; on the one hand, people are complaining that rules are too complex and don’t make sense, while on the other, the same people are claiming that they have the skills and intelligence to determine for themselves which risks to take during a global pandemic. The flipside of this irony is that it is the people who understand the logic of the rules who have are most likely to have the skills and least likely to want to do their own risk assessment: they understand the importance of respecting the rules.

Perhaps the core problem is that the people whose lives are saved by compliance are very morally distant from the people who need to comply; this is a problem in itself but it also makes it seem even ruder to challenge someone to obey the rules. If a rule makes sense, people can easily see how not following it could infect someone; but if a regulation or rule seems unfair, or arbitrary, that increases the moral distance from those who might be killed by the virus in a few weeks, as there won’t seem to be any causal link between the two.

## Conclusion

In the initial months of lockdown, the rules were simple and almost everyone obeyed them. Society has opened up since and is now closing down again because the new rules regulating how we should behave were not followed, either because they were too complex or because people relaxed too much or both. If we are to avoid a third wave, rules will remain important, and so will the obligation to get others to follow them. But anyone brave enough to remind rulebreakers of the rules may find it difficult to get others to follow them: they may be perceived as rude, condescending, and interfering, even if rulebreakers are risking everyone’s rude health. The role of social distance warrior is more important than ever because of increasing non-compliance due to perceived arbitrariness of rules, but also more difficult than ever, for precisely the same reason.

## References

[CR1] Benham, J.L., R. Lang, K. Kovacs Burns, et al. Attitudes, current behaviours and barriers to public health measures that reduce COVID-19 transmission: A qualitative study to inform public health messaging. *PLOS One* 2021: 10.1371/journal.pone.0246941 10.1371/journal.pone.0246941 PMC7895406 33606782

[CR2] BBC. Wales coronavirus rules: No more than six people can meet indoors but under-11s don't count. September 12, 2020. https://www.bbc.co.uk/newsround/54113993. Accessed September 22, 2021.

[CR3] Iacobucci G (2020). Sixty seconds on . . . stay alert. BMJ.

[CR4] Scottish Government. 2020a. Remember FACTS for a safer Scotland. 7^th^ July 2020. https://www.gov.scot/binaries/content/documents/govscot/publications/advice-and-guidance/2020/08/coronavirus-covid-19-facts-poster-translations/documents/english/english/govscot%3Adocument/20-21%2B-%2BCoronavirus%2B-%2BTranslations%2B-%2BFACTS%2BPoster%2B-%2BEnglish%2B-%2B9%2BJuly%2B2020.pdf. Accessed MMMM DD, YYYY.

[CR5] ———. 2020b. Maximum gathering set at six people from two households. September 10. https://www.gov.scot/news/maximum-gathering-set-at-six-people-from-two-households/. Accessed September 22, 2021.

[CR6] Shaw, D. 2020a. The UK government is encouraging people outside England to break lockdown rules. *Journal of Medical Ethics Blog*, May 20. https://blogs.bmj.com/medical-ethics/2020/05/20/3835/. Accessed September 22, 2021.

[CR7] ———. 2020b. The many meanings of “stay safe” in a pandemic: Sympathy, duty, and threat. *Journal of Medical Ethics Blog*, May 13. https://blogs.bmj.com/medical-ethics/2020/05/13/the-many-meanings-of-stay-safe-in-a-pandemic-sympathy-duty-and-threat/. Accessed September 22, 2021.

[CR8] Traynor, S. 2020. Coronavirus in Scotland: Indoor gatherings to be banned from Friday under new restrictions. September 22. https://www.edinburghlive.co.uk/news/uk-world-news/coronavirus-scotland-indoor-gatherings-banned-18977577. Accessed September 22, 2021.

[CR9] U.K. Government. 2020a. Guidance on staying at home and away from others. March 23 (withdrawn May 11, 2020). https://www.gov.uk/government/publications/full-guidance-on-staying-at-home-and-away-from-others. Accessed September 22, 2021.

[CR10] ———. 2020b. Get a discount with the Eat Out to Help Out Scheme. July 15 (withdrawn August 31, 2020). https://www.gov.uk/guidance/get-a-discount-with-the-eat-out-to-help-out-scheme. Accessed September 22, 2021.

[CR11] ———. 2020c. New campaign to prevent spread of coronavirus indoors this winter (press release). September 9. https://www.gov.uk/government/news/new-campaign-to-prevent-spread-of-coronavirus-indoors-this-winter. Accessed September 22, 2021.

[CR12] ———. 2020d. Rule of six comes into effect to tackle coronavirus. September 14. https://www.gov.uk/government/news/rule-of-six-comes-into-effect-to-tackle-coronavirus Accessed September 22, 2021.

